# Evaluation of the impact of single-nucleotide polymorphisms on treatment response, survival and toxicity with cytarabine and anthracyclines in patients with acute myeloid leukaemia: a systematic review protocol

**DOI:** 10.1186/s13643-019-1011-y

**Published:** 2019-05-03

**Authors:** Taynah Cascaes Puty, Jonathan Souza Sarraf, Tabata Cristina Do Carmo Almeida, Valter Cordeiro Barbosa Filho, Luis Eduardo Werneck de Carvalho, Fernando Luiz Affonso Fonseca, Fernando Adami

**Affiliations:** 1 Ensino e Pesquisa, Oncológica do Brasil Ensino e Pesquisa, Belém, Brazil; 2 Laboratório de Epidemiologia e Análise de Dados, Faculdade de Medicina do ABC, Santo André, Brazil; 3 Universidade Federal do Pará, Belém, Brazil; 4 Faculdade de Ciências Naturais, ICEN, UFPA, Belém, Brazil; 5 Federal Institute of Education, Science and Technology of Ceara, Boa Viagem Campus, Boa Viagem, Brazil

**Keywords:** SNP, Standard chemotherapy, Acute myeloid leukaemia, Survival, Toxicity

## Abstract

**Introduction:**

Acute myeloid leukaemia is the most common type of acute leukaemia in the world. Thus, the study of genetic alterations, such as single-nucleotide polymorphisms (SNPs), has contributed to a better understanding of the mechanisms underlying leukaemogenesis, to improve the prognosis and to increase the survival of these patients. However, there is no synthesis of evidence in the literature evaluating the quality of evidence and the risk of bias in the studies such that the results can be translated. Thus, this systematic review protocol aims to assess the impact of SNPs on genes involved in the metabolism of cytarabine and anthracyclines with respect to survival, treatment response and toxicity in patients with AML.

**Methods:**

This systematic review protocol is based on PRISMA guidelines and includes searches in six electronic databases, contact with authors, repositories of clinical trials, and cancer research. Studies published in peer-reviewed journals will be included if they meet the eligibility criteria: (a) samples composed of individuals of any age, of both sexes, with a diagnosis of AML, regardless of the time of diagnosis of disease; (b) participants who have undergone or are undergoing cytarabine- and anthracycline-associated chemotherapy or cytarabine-only chemotherapy; and (c) in vivo studies. Studies that include patients with promyelocytic leukaemia (Fab type 3) will be excluded because this disease has different treatment. The process of study selection, data extraction, and evaluation/synthesis will be performed in duplicate. Assessment of methodological quality and risk of bias will be performed using the Cochrane Risk of Bias Tool for randomized clinical studies and the Downs-Black Checklist for cohort and case-control studies. The synthesis of evidence will include the level of evidence based on the GRADE protocol. A meta-analysis of the association between SNPs and outcomes may be performed based on Cochrane guidelines.

**Discussion:**

It is expected that clinical decisions for AML patients will consider evidence-based practices to contribute to better patient management. In this way, we will be able to define how to treat patients with AML to improve their survival and quality of life.

**Systematic review registration:**

PROSPERO CRD42018100750

**Electronic supplementary material:**

The online version of this article (10.1186/s13643-019-1011-y) contains supplementary material, which is available to authorized users.

## Background

Acute myeloid leukaemia (AML) is a malignant and heterogeneous [1] haematopoietic disease [2] characterized by an abnormal growth of proliferative, clonal and highly differentiated white blood cells [3, 4]. These cells infiltrate the bone marrow and stimulate the production of progenitor or abnormal white cells, preventing the system from maturing its cells to perform adequate defence functions in the organism [5], and infiltrate other tissues, which can lead to relapse and an increased risk of death [6].

AML is the most common type of acute leukaemia in the world [7], with an estimated worldwide prevalence of 160 cases and an incidence of 103 new cases per 100,000 inhabitants in 2016 [8]. In addition, the number of deaths worldwide in 2012 due to leukaemia was 265,471 [9]. In the USA, the estimated 5-year survival rate is 26.9% [10]. Thus, the study of genetic alterations related to the appearance of AML, such as somatic mutations and single-nucleotide polymorphisms (SNPs), has contributed to a better understanding of the mechanisms underlying leukaemogenesis, to improve prognosis [3, 11–13] and to increase the survival of these patients [2, 5].

AML is treated by a combination of cytarabine with an anthracycline (daunorubicin or idarubicin) [14], resulting in a remission rate of approximately 80% [7, 15]. Cytarabine (Ara-C) is an analogue of deoxycytidine [16], which blocks the synthesis of DNA [11, 15, 17]. Cytarabine metabolism starts in the cell membrane. This is because the SLCO1B1 and SLC29A1 genes encode membrane transporters that transport Ara-C to the intracellular space [15, 18, 19]. Subsequently, the DCK gene [20] encodes the enzyme that catalyzes the rate-limiting first phosphorylation step in the activation of cytarabine to cytarabine monophosphate (Ara-CMP). The NT5C3A gene acts opposite to the DCK gene by reactivating cytarabine [21]. The gene CDA [22, 23] is the main gene that promotes the inhibition of the drug through the production of an enzyme that causes irreversible deamination (Ara-U) [15]. The genes RRM1, RRM2 and RRM2B are responsible for producing enzymes that transform cytidine triphosphate (CTP) to deoxycytidine diphosphate (dCDP) [24], which is transformed to deoxycytidine triphosphate, which enters the cell nucleus to block DNA synthesis [15, 25, 26].

Anthracyclines are metabolized in various tissues. Metabolism also begins in the cellular membrane, for example, by the gene SLC22A12 [27]. Cation-anion transport generates the influx of the drug into cells. Another transporter involved is ABCB1, which belongs to the ABC family and is associated with cellular efflux [15]. The transformation of the drug to semiquinone is performed by several oxidoreductases, including NOS3, which is a nitric oxide synthase, and is related to the decrease in enzymatic activity [15, 27].

Genes related to the metabolism of gold standard drugs have SNPs, which are related to the outcomes related to the treatment of AML [15, 26]. For example, some SNPs (e.g. rs2306744, rs80143932, rs1561876, rs2898950, rs3750117, rs1265138, rs1045642, rs2032582, rs1128503) have been associated with better or worse response to treatment, depending on the genotypes present. On the other hand, other SNPs (e.g. rs4149056, rs532545, rs2072671, rs11231825) have been related to higher or lower toxicity or to overall and/or disease-free survival (e.g. rs2291075, rs1042919, rs1130609, rs5030743, rs2032582, rs1799983) [15].

Studies on the influence of SNPs on the outcomes of AML have focused on aspects such as survival (SLD and SG) [28, 29], treatment response [30] and toxicity [11, 15, 27, 31]. There is a database registered under HHS and financially supported by NIH/NIGMS. This database collects, curates and disseminates data on the impact of genetic variations in humans on drug responses. This registry was made by annotating genetic variants and gene-drug-disease relationships via literature review and summarizing important pharmacogenomic data and associations between genetic variants and drugs, and drug pathways, among others [26].

However, the findings in the literature are still insufficient regarding the evaluation of the level of evidence and the risk of bias in studies that address this issue. In addition, there is no other similar review under development registered at PROSPERO [32]. Therefore, a study that fills these gaps will be important to translate the results into recommendations for clinical practice based on reliable evidence [15, 26].

Therefore, this study aims to evaluate the impact of SNPs on treatment response, survival and toxicity with cytarabine and anthracyclines in AML patients. Consequently, the study outlines a systematic review protocol that seeks to address this research question. Thus, recommendations and guidelines for interventions in AML patients are expected to rely more on evidence-based practices to contribute to better patient management. In this way, we will know how to improve the survival and quality of life in these patients.

## Methods

### Study registration

The protocol for this systematic review is based on the Preferred Reporting Items for Systematic Reviews and Meta-Analyses Protocols (PRISMA-P) (Additional file 1) [33], as well as the Cochrane Handbook for Systematic Reviews of Interventions—Cochrane Book Series [34].

This protocol is registered under the International Prospective Register of Systematic Reviews (PROSPERO) (registration number CRD42018100750).

### Identification of eligible studies

Included in this review will be studies published in peer-reviewed journals that meet the following eligibility criteria, organized by population, intervention/exposure, control, outcome and study design (PICOS framework) [35, 36].

#### Population

Studies will be accepted (a) when the sample is composed of individuals of any age, of both sexes, with a diagnosis of acute myeloid leukaemia, and independent of the time of diagnosis of the disease; (b) when participants who have undergone or are undergoing cytarabine- and anthracycline-associated [11, 14] or isolated cytarabine chemotherapy—the latter being the main and earliest chemotherapy used for AML [29, 37]; and (c) when they are in vivo. Studies that include patients with promyelocytic leukaemia (Fab type 3) will be excluded because other types of treatment are used for this condition [15].

#### Intervention/exposure

Single-nucleotide polymorphism is a type of polymorphism characterized by a single-nucleotide variation in a genetic sequence occurring in a minimum of one population [38]. In AML, there are several SNPs, but not all of them are related to the metabolism of cytarabine and anthracyclines [15, 30] or influence disease-free survival, overall survival, treatment response (ORR), complete response (CR), complete response with incomplete blood recovery (CRi) [28] or toxicity [15, 26, 31].

To select the SNPs to be studied, we used the PharmGKB database (https://www.pharmgkb.org). PharmGKB® is registered under HHS and is financially supported by NIH/NIGMS. It is managed at the Stanford University (R24 GM61374). It has Clinical Pharmacogenetics Implementation Consortium (CPIC®), Pharmacogenomics Research Network (PGRN), precisionFDA and PharmCAT as partners. Clinical Pharmacogenetics Implementation Consortium (CPIC®) started as a shared project between PharmGKB and Pharmacogenomics Research Network (PGRN) in 2009. CPIC guidelines are indexed in PubMed as clinical guidelines and referenced in ClinGen and PharmGKB [26].

After searching the database, the data were compared with a review manuscript evaluating polymorphisms that influence the treatment of AML [15]. The descriptions of all genes and their SNPs were initially evaluated on the PharmGKB database to assess whether they are related to cytarabine and anthracycline metabolism (daunorubicin or idarubicin). The articles and the PharmGKB database were then compared, and the SNPs that appeared in both references were considered related to drug metabolism. All SNPs with confirmed relationship were selected. The final compilation of genes identified in these analyses included the following SNPs: SLCO1B1 (rs2291075 [18, 19] and rs4149056); DCK (rs2306744 [20] and rs80143932); RRM1 (rs1042919, rs1561876 and rs2898950); RRM2 (rs1130609 and rs5030743); RRM2B (rs1265138) [24]; NT5C3A (rs3750117) [21]; CDA (rs532545 and rs2072671) [22, 23]; ABCB1 (rs1045642, rs2032582 and rs1128503); SLC22A12 (rs11231825); and NOS3 (rs1799983). In addition, other SNPs found in the search will be evaluated, as long as they are related to the outcomes, taking into account the inclusion criteria. The influence of these SNPs on the outcomes is presented in Additional file 2.

#### Outcomes

Overall survival and disease-free survival will be selected as outcomes according to the Food and Drug Administration (FDA) [37]. The outcome of toxicity to treatment will be checked using the type of toxicity/adverse event and grade [27, 31]. Response to treatment will be assessed by means of ORR, CR and CRi [28].

#### Primary outcomes

1. Toxicity [27, 31]

- Type (e.g. anaemia, leukopenia, neutrophilia)
- Grades 1 to 5

2. Prognostic outcomes [37]

- Overall survival
- Disease-free survival

3. Response to treatment

- Overall response rate (ORR)
- Complete response (CR)
- Complete response with incomplete blood recovery (CRi)
- Partial response
- Drug resistance

#### Type of study

If found, randomized controlled trials (RCTs) that meet the following criteria will be included: containing at least one group evaluating the SNPs studied, assessing how these SNPs modify the metabolism of cytarabine and anthracyclines or how SNPs affect the outcomes of interest and using at least cytarabine in all arms. Additionally, we will include cohort and case-control studies evaluating any of these SNPs, regardless of the follow-up time and direction of study (retrospective or prospective for cohort studies). We will not include cross-sectional observational studies, as it is not possible to perform prognostic evaluation through this type of study. Furthermore, we will include manuscripts published in peer-reviewed journals, without assigning limits to date or language of publication.

### Search strategy

An electronic search to identify publications that meet the selection criteria will be carried out in five databases and one repository of studies.

Keywords will be selected according to medical subject headings (MeSH) in the National Library of Medicine and text relevant to the area. The organization of terms for the study will be carried out according to the PICOS framework. To improve the search for articles, the following databases and strategies will be used

MEDLINE (Additional file 3): used with MeSH and all their *entry terms*;

1. Scopus (Additional file 4);
2. Web of Science (Additional file 5);
3. Cochrane Clinical Trials (Additional file 6);
4. EMBASE (Additional file 7);
5. PharmGKB—repository of studies.

In addition to database searches, the following search strategies will be employed:

i) *Consultation of the reference lists of all original articles included*: After the final selection, we will review references to identify clinical trials that may not have been found in the initial search.
ii) *Contact with authors*: (1) if complete articles are not available; (2) after article study selection, authors will be consulted about other publications on the subject that may not have been found in the initial search; and (3) if certain data are not available in the original article, such as data presented only in graphs.
iii) *Searches in repositories of clinical trials and cancer research*: We will also consult ClinicalTrials.gov (http://clinicaltrials.gov/), repository of cancer studies (https://www.unmc.edu/cancercenter/registry/) and Brazilian Clinical Trials Registry (http://www.ensaiosclinicos.gov.br). For ClinicalTrials.gov and the Cancer Center registry, we will use the term “*acute myeloid leukaemia and polymorphism (or genetic polymorphisms or single-nucleotide or SNP or pharmacogenetics)*”. For the search in the Brazilian registry, the term “acute myeloid leukaemia and polymorphism (or genetic polymorphisms or single-nucleotide or SNP or pharmacogenetics)” will be used. Eligibility criteria will be applied to the original studies and those in the repositories for inclusion in this review. If we find eligible studies that are unpublished, we will contact the authors to obtain the results.

### Study records

#### Data management

The management of references and removal of duplicates will be performed with EndNote X7, and articles will be managed using Excel spreadsheets and Review Manager (RevMan) software (http://community.cochrane.org/tools/review-production-tools/revman-5).

#### Selection process

After the completion of search in the proposed databases, a single library will be created, and duplicates will be removed. After this process, two reviewers will independently select articles. There will be two levels to the selection process. At the first level, reviewers will screen articles by reading titles and abstracts according to the defined inclusion criteria. The reviewers must justify the exclusion of any items in the spreadsheet.

At the second level of selection, the reviewers will read full texts. At this stage, the reviewers will evaluate the defined inclusion criteria. Exclusions will be justified in a spreadsheet, and a third reviewer will be used to resolve disagreements.

After each two-step selection, a consensus meeting will be held to review which articles will be considered eligible for review. If necessary, a third reviewer will be used to resolve disagreements.

After the selection process in electronic databases, complementary search strategies (author contact and reference list screening) will be employed by one reviewer to identify additional studies.

### Study synthesis and analysis

All stages of data extraction, management, risk of bias, evidence level and synthesis will be independently implemented by two reviewers. A third reviewer will resolve disagreements, if necessary.

#### Data extraction

Reviewers will receive the following spreadsheet (elaborated by TCP) in Excel format with all the variables to be filled by them. Descriptive results will be presented according to Table 1 (Additional file 8).

**Table 1:** Characteristics and variables to be extracted from the included articles

Characteristics	Variables
Study characteristics	Reference; study type; country; context
Population	Participants number; amount of groups; age in years; eligibility criteria; sample selection method; treatment performed; type of AML
Exposures	SNP; DNA localization; related gene; altered nitrogen bases
Outcomes	Toxicity (degree and type); disease-free and global survival; response to treatment
Results	Description of the data; confidence intervals and *p* values of the study, survival and treatment response in randomized clinical trials; assessment of outcomes in cohort and case-control studies

#### Risk of bias and methodological quality of individual studies

The risk of bias will be individually assessed for all studies using the Cochrane Risk of Bias Tool [39] for randomized clinical trials and the Downs-Black Checklist [40] for observational cohort and case-control studies.

The Cochrane Risk of Bias Tool for randomized controlled trials [39] evaluates patient selection, biased allocation, publication of selective and incomplete results and blinding of participants and researchers to assess whether the criteria used have a low risk, high risk or unclear risk of bias. In addition, this tool evaluates the possibility of other forms of bias, such as fraud or other problems.

The Downs-Black Checklist [40] contains 27 items for the evaluation of information quality, internal validity (bias and confounders), study power and external validity. All items are coded as 0 and 1 (0 representing worse quality), except for the question (“Are the distributions of the main confounders in each group of subjects to be compared to clearly described?”), which is coded as 0, 1 or 2. Thus, at the end of the evaluation, the studies will be ranked from 0 to 28, with 0 indicating the worst quality and 28 indicating the best quality.

#### Data synthesis

The authors will consider whether to perform a meta-analysis on the impact of each SNP on treatment with cytarabine and anthracyclines regarding each outcome for this review. The decision on how to group the studies in the meta-analysis will be based on their characteristics (PICOS elements) to guarantee clinical homogeneity between the studies. Studies with insufficient data for the meta-analysis will be retained in the systematic review. If the authors decide not to perform the meta-analysis or if it is not feasible because of the quantity, quality or heterogeneity of the studies, the data will be presented as a narrative synthesis.

Homogeneity for clinical and statistical questions will be considered in deciding whether a meta-analysis is performed. For this, data extraction and effect size estimation will be performed for each prognostic outcome and each SNP, which include continuous (e.g. the means and standard errors for the mean in exposed and non-exposed groups or the mean difference between groups) or categorical effect measure (e.g. the overall event rate of a time-to-event outcome across the whole study period or hazard ratio). Adjusted effect measures will be preferred over unadjusted measured. A review on the performance of a certain prognostic model would extract measures such as discrimination (c-statistic) and calibration (calibration slope, OE ratio) and, if applicable, reclassification (NRI), net benefit measures, etc. For predictive/treatment selection factor studies, the key statistic to extract will be the treatment-covariate interaction estimate; that is, the estimated difference in treatment effect according to changes in a particular predictor (covariate). Formulae for continuous (mean difference) and categorical (relative risk) outcomes will be applied to estimate the effect size (and 95% confidence interval (95% CI)) using Review Manager. If there is insufficient information to perform effect-size calculations, the mean values and/or standard deviations will be requested from the authors of the original studies. Variability in the effect of the interventions will be tested for statistical heterogeneity using the chi-square (*χ*^2^) test with the corresponding *p* value (Cochrane test) and *I*^2^ statistic. Heterogeneity will be considered low if *I*^2^ ≤ 50%. The level of significance will be set at 5%, and all analyses will be performed in RevMan. For low heterogeneity, a fixed-effect meta-analysis will be used to estimate the treatment effect; for high heterogeneity, we will use a random-effects model.

Subgroup analyses may be performed, considering the clinical and statistical aspects, to identify factors acting as moderators of the intervention. These analyses will be performed to determine whether factors, such as adequate sample size, were addressed by identifying these factors, and a sensitivity analysis of the synthetic measurements will be performed. Although it is not possible to predict all of these adjustments in advance, we believe that the factors related to different SNPs can be used in sensitivity analyses depending on the number of articles included.

The certainty of evidence across studies will be evaluated according to the GRADE protocol [41–43] for each outcome considering each SNP. We will estimate the evidence level according to Grades of Recommendation, Assessment, and Development and Evaluation [42]. This tool allows for the definition of four evidence levels (high, moderate, low or very low). The study design is the starting point in assessing the quality of evidence for each outcome. Randomized controlled trials are designated with the highest level of evidence because they are considered to be less prone to methodological limitations. Observational studies will be initially considered to provide the lowest level of evidence because they have greater methodological limitations. There are five possible factors that subsequently diminish study quality: risk of bias (or methodological limitations), inconsistency, imprecision, indirect evidence and publication bias [34].

In the presentation of data, the results will be first organized in alphabetical order by the main authors; if the first author is repeated, we will organize the articles in chronological order by the year of publication. All forest plots will also be grouped by the type of study.

In the case of modification of the protocol in the completed publication of the results of this systematic review, the authors will clarify and justify all modifications in a specific section.

## Discussion

AML is the most common type of leukaemia in the world [7]; hence, many studies have attempted to evaluate better forms of AML treatment. However, to the best of our knowledge, no systematic review has attempted to synthesize the level and quality of evidence on how genetic modifications impact survival, treatment response and toxicity in AML [12, 13, 15, 29]. In particular, it is necessary to synthesize evidence from the literature about the impact of SNPs on the evaluated outcomes and to consider aspects of the methodological quality of and the level of evidence in the studies. This is necessary for establishing recommendations and guidelines for effectively and safely using these interventions in clinical practice, considering the different effects expected from their use in patients with AML [15, 26].

Thus, this protocol will provide robust evidence for searches and future studies on the effect SNPs on treatment in patients with AML, thereby presenting and categorizing the existing evidence according to different expected outcomes (e.g. creating a profile that represents greater toxicity or better response), according to the methodological quality, generalization capacity and risk of bias of studies. Moreover, unlike the protocols proposed thus far, the objective of this protocol is to expand the reach of literature search, using databases, clinical trial repositories, and contact with authors, to identify studies evaluating a range of SNPs and not just the influence of a single SNP on a single prognostic endpoint. Thus, we will be able to evaluate the real influence of SNPs, both individually and combined, on the metabolism of chemotherapy drugs used in the standard treatment of AML and on several prognostic outcomes.

Among the limitations of the study will be the large number of SNPs that can be present (apart from those already evaluated) and the great variety of the types and methodologies of studies carried out. However, categorization of the types of studies, SNPs, drugs used and outcomes may help in the search and evaluation of the subgroups generated. Therefore, we can define the subgroups to use in classifying the available evidence (to apply in clinical practice) and the subgroups that will need more studies to define the real clinical effect.

With the results of this systematic review, clinical decisions for AML patients may be expected to rely on evidence-based practices, thus contributing to better patient management. In this way, it will be possible to evaluate where we should invest in larger studies to better define patient profiles that may, for example, respond better or require a lower dose because of higher toxicity. Thus, we will be able to better define how to treat patients with AML to improve their survival and quality of life.

## Additional files

Additional file 1: PRISMA-P checklist. (PDF 473 kb)

Additional file 2: Genes and drugs. (DOCX 25 kb)

Additional file 3: MEDLINE database. (DOCX 19 kb)

Additional file 4: Scopus database. (DOCX 16 kb)

Additional file 5: Web of Science database. (DOCX 16 kb)

Additional file 6: Cochrane Clinical Trials database. (DOCX 17 kb)

Additional file 7: EMBASE database. (DOCX 15 kb)

Additional file 8: Data dictionary for extract data. (DOCX 27 kb)
