# Supplementary material for: Evaluation of the impact of single-nucleotide polymorphisms on treatment response, survival and toxicity with cytarabine and anthracyclines in patients with acute myeloid leukaemia: a systematic review protocol
Source: Syst Rev. 2019 May 3;8:109. doi: 10.1186/s13643-019-1011-y (PMC6499963; doi:10.1186/s13643-019-1011-y)
Supplement: Supplementary file 2 — Genes and drugs. (DOCX 25 kb) [file 13643_2019_1011_MOESM2_ESM.docx]

**Additional file 2 –** Relation between genes and drugs by PharmGKB

| **Gene** | **Variant** | **Class** | **Drug** | **Alleles** | **Function** | **Evidence** | **effect** |
| --- | --- | --- | --- | --- | --- | --- | --- |
| **SLCO1B1** | [rs2291075](https://www.pharmgkb.org/variant/PA166154544) | Synonymous | cytarabine, daunorubicin, etoposide | C > T | SNP | level 3 | Event-free survival and Overall survival: CT/TT>CC |
|  | [rs4149056](https://www.pharmgkb.org/variant/PA166154579) | Missense | cytarabine | T > C | SNP | level 3 | Toxic liver disease: CC>CT>TT |
| **DCK** | [rs2306744](https://www.pharmgkb.org/variant/PA166156554) | 5 UTR | cytarabine | C > T | SNP | level 3 | Treatment response: TT>CT>CC |
|  | [rs80143932](https://www.pharmgkb.org/variant/PA166156697) | 5 Flanking | cytarabine | C > G | SNP | level 3 | Treatment response: GG>GC>CC |
| **RRM1** | [rs1042919](https://www.pharmgkb.org/variant/PA166154310) | 3 UTR | cytarabine | A > T | SNP | level 3 | Event-free survival : AA>AT |
|  | [rs1561876](https://www.pharmgkb.org/variant/PA166154332) | 3 UTR | cytarabine | G > A | SNP | level 3 | Treatment response: AA/AG>GG |
|  | [rs2898950](https://www.pharmgkb.org/variant/PA166154392) | Intronic | cytarabine | A > C | SNP | level 3 | Tratment response: AC/CC>AA |
| **RRM2** | [rs1130609](https://www.pharmgkb.org/variant/PA166155627) | 5 UTR | cytarabine | T > G | SNP | level 3 | Overall survival: GG>GT/TT |
|  |   rs5030743 | Synonymous | cytarabine | C > G | SNP | level 3 | Overall suvival: CC>CG/GG |
| **NT5C3A** | [rs3750117](https://www.pharmgkb.org/variant/PA166157378) | Synonymous | cytarabine | A > G | SNP | level 3 | Treatment response: AA/AG>GG |
| **RRM2B** | [rs1265138](https://www.pharmgkb.org/variant/PA166157554) | Unannotated | cytarabine | A > G | SNP | level 3 | Treatment response: AA>AG/GG |
| **CDA** |   rs532545 | 5 Flanking | cytarabine | C > T | SNP | level 3 | Toxicity: CT/TT>CC |
|  | rs2072671 | Missense | cytarabine | A > C | SNP | level 3 | Toxicity: CC/AC >AA |
| **ABCB1** | rs1045642 | Synonymous | antrracyclines, cytarabine | A > G | SNP | level 3 | treatment response: AA>AG/GG |
|  | rs2032582 | Missense | cytarabine, daunorubicin | A > C; A > T | SNP | level 3 | Treatment response: AA>AC/AT> CC/TT |
|  |   rs1128503 | Synonymous | cytarabine | A > G | SNP | level 3 | Treatment response: AA/AG>GG |
| **SLC22A12** | rs11231825 | Synonymous | cytarabine | T > C | SNP | level 3 | Fever Events: TT>CT/TT |
| **NOS3** | rs1799983 | Missense | daunorubicin | T > G | SNP | level 3 | Overall suvival: GG>GT/TT |
