# Supplementary material for: Evaluation of the impact of single-nucleotide polymorphisms on treatment response, survival and toxicity with cytarabine and anthracyclines in patients with acute myeloid leukaemia: a systematic review protocol
Source: Syst Rev. 2019 May 3;8:109. doi: 10.1186/s13643-019-1011-y (PMC6499963; doi:10.1186/s13643-019-1011-y)
Supplement: Supplementary file 3 — MEDLINE database. (DOCX 19 kb) [file 13643_2019_1011_MOESM3_ESM.docx]

**Additional file 3 –** Description of the search terms according to the Medline database (PUBMED)

|  | **Data base**: Medline  **Descriptors** |
| --- | --- |
| **#1** | (Acute Myeloid leukaemi*) **OR** (Acute Myeloid leukemi*) **OR** “ANLL” **OR** (Leukaemia, Acute Myelogenous*) **OR** “Leukaemia, Acute Myeloid” **OR** “Leukaemia, Acute Myeloblastic” **OR** “Leukaemia, Acute Myelocytic” **OR** “Leukaemia, Acute Nonlymphoblastic” **OR** “Leukaemia, Acute Nonlymphocytic” **OR** “Leukaemias, Acute Myelogenous” **OR** “Leukaemias, Acute Myeloblastic” **OR** “Leukaemias, Acute Myelocytic” **OR** “Leukaemias, Acute Nonlymphoblastic” **OR** “Leukaemias, Acute Nonlymphocytic” **OR** “Myeloid Leukaemia, Acute, M1” **OR** “Acute Myeloid Leukaemia without Maturation” **OR** “Myeloid Leukaemia, Acute, M2” **OR** “Acute Myeloid Leukaemia with Maturation” |
| **#2** | (Single nucleotide polymorphism*) **OR** “SNPs” **OR** “rs2291075” **OR** “rs4149056” **OR** “[rs2306744](https://www.pharmgkb.org/variant/PA166156554)” **OR “**rs1042919” **OR “**rs1561876” **OR** “rs1130609” **OR “**rs3750117” **OR “**rs532545” **OR** “rs2072671” **OR** (Solute Carrier Organic Anion Transporter Family Member 1b1*) **OR** (SLC21A6 Transporter*) **OR** (LST-1 Transport Protein*) **OR** (Organic Anion Transport Polypeptide C*) **OR (**Oatp C Transport Protein*) **OR “**SLCO1B1 Protein” **OR (**Organic Anion Transport Polypeptide 2) **OR** (Deoxycytidine Kinase*) **OR** “DCK” **OR** (ribonucleotide reductase M1 polypeptide) **OR** “ribosomal reductase M2” **OR** (ribonucleotide reductase M2 subunit*) **OR “**RRM2 protein” **OR (**ribonucleotide reductase M2 polypeptide) **OR** “ribonucleotide reductase M2 B (TP53 inducible) protein” **OR (**p53-inducible ribonucleotide reductase small subunit 2*) **OR “**NT5C3A” **OR (**Cytidine Deaminas*) **OR** “CDA” **OR** “ATP-Binding Cassette, Sub-Family B, Member 1” **OR** (P Glycoprotein*) **OR** (PGY 1 Protein*) **OR** “Multidrug Resistance Protein 1” **OR** “ABCB1 Protein” **OR** “MDR1 Protein” **OR** “rs1045642” **OR** “rs2032582” **OR** “rs1128503” **OR** “SLC22A12 protein” **OR (**urate transporter 1 protein *) **OR** “organic anion transporter 4 like protein” **OR** (solute carrier family 22 organic anion cation transporters, member 12 protein*) **OR** “rs11231825” **OR (**NOS3 protein*) **OR** “nitric oxide synthase 3, endothelial cell protein, human” **OR** “ECNOS protein” **OR** “rs1799983” **OR** (Cytochrome P 450 CYP2E1*) **OR** (Cytochrome P 450 J*) **OR** (4 Nitrophenol 2 Hydroxylase*) **OR** (Dimethylnitrosamine N Demethylase*) **OR** “CYP 2E1” **OR** (Cytochrome P 450 IIE1*) **OR** (CYPIIE1*) **OR** “Cytochrome P-450 (ALC)” **OR** “CYP2E1” **OR** “rs2070673” **OR** “rs2515641” |
| **#3** | (randomized controlled trial*) [Publication Type] **OR**  “controlled clinical trial” [Publication Type] **OR** “random allocation” [MeSH Terms] **OR** “double blind method” [MeSH Terms] OR “single blind method” [MeSH Terms] **OR** “clinical trial” [Publication Type] **OR** "cohort studies"[MeSH Terms] **OR** “Concurrent Studies” **OR** “Closed Cohort Studies” **OR** “Cohort Analysis” **OR** “Historical Cohort Studies” **OR** “case-control studies"[MeSH Terms] **OR** “Case-Control Study” **OR** “Case Comparison Studies” **OR** “Case-Compeer Study” **OR** “Case-Referrent Study” **OR** “Case Referrent Studies” **OR** “Case-Referrent Study” **OR** (Case-Base Studies*) **OR** (Nested Case-Control Study*) **OR** (Matched Case-Control Study*) |
| **#4** | (disease-free survival*)**OR** (Event-Free Survival*) **OR** (Progression-Free Survival*) **OR** " overall survival " **OR** “Drug Related Side Effects and Adverse Reactions” [MeSH Terms] **OR** “Adverse Drug Event” **OR** “Adverse Drug Events” **OR** “Drug Event, Adverse” **OR** “Drug Events, Adverse” **OR** “Side Effects of Drugs” **OR** “Drug Side Effects” **OR** “Drug Side Effect” **OR** “Effects, Drug Side” **OR** “Side Effect, Drug” **OR** “Side Effects, Drug” **OR** “Adverse Drug Reaction” **OR** “Adverse Drug Reactions” **OR** “Drug Reaction, Adverse” **OR** “Drug Reactions, Adverse” **OR** “Reactions, Adverse Drug” **OR** “Drug Toxicity” **OR** “Toxicity, Drug” **OR** “Drug Toxicities” **OR** “Toxicities, Drug” **OR** “overall response rate” **OR** “complete response” **OR** “complete response with incomplete blood recovery” |
| **#5** | **#1** AND **#2** AND **#3** AND **#4** |
| **#6** | **Limits:** Species (humans), language (English); without limitation of age or year of publication. |
