# Supplementary material for: Evaluation of the impact of single-nucleotide polymorphisms on treatment response, survival and toxicity with cytarabine and anthracyclines in patients with acute myeloid leukaemia: a systematic review protocol
Source: Syst Rev. 2019 May 3;8:109. doi: 10.1186/s13643-019-1011-y (PMC6499963; doi:10.1186/s13643-019-1011-y)
Supplement: Supplementary file 4 — Scopus database. (DOCX 16 kb) [file 13643_2019_1011_MOESM4_ESM.docx]

**Additional file 4** - Description of the search terms according to the Scopus database.

|  | **Data base**: Scopus  **Descriptors** |
| --- | --- |
| **#1** | TITLE-ABS-KEY ((Acute Myeloid Leukaemi*) OR (Acute Myeloid Leukemi*) OR “ANLL” OR (Leukaemia, Acute Myelogenous*) OR “Leukaemia, Acute Myeloid” OR “Leukaemia, Acute Myeloblastic” OR “Leukaemia, Acute Myelocytic” OR “Leukaemia, Acute Nonlymphoblastic” OR “Leukaemia, Acute Nonlymphocytic” OR “Leukaemias, Acute Myelogenous” OR “Leukaemias, Acute Myeloblastic” OR “Leukaemias, Acute Myelocytic” OR “Leukaemias, Acute Nonlymphoblastic” OR “Leukaemias, Acute Nonlymphocytic” OR “Myeloid Leukaemia, Acute, M1” OR “Acute Myeloid Leukaemia without Maturation” OR “Myeloid Leukaemia, Acute, M2” OR “Acute Myeloid Leukaemia with Maturation”) |
| **#2** | TITLE-ABS-KEY((Single nucleotide polymorphism*) **OR** “SNPs” **OR** “rs2291075” **OR** “rs4149056” **OR** “[rs2306744](https://www.pharmgkb.org/variant/PA166156554)” **OR “**rs1042919” **OR “**rs1561876” **OR** “rs1130609” **OR “**rs3750117” **OR “**rs532545” **OR** “rs2072671” **OR** (Solute Carrier Organic Anion Transporter Family Member 1b1*) **OR** (SLC21A6 Transporter*) **OR** (LST-1 Transport Protein*) **OR** (Organic Anion Transport Polypeptide C*) **OR (**Oatp C Transport Protein*) **OR “**SLCO1B1 Protein” **OR (**Organic Anion Transport Polypeptide 2) **OR** (Deoxycytidine Kinase*) **OR** “DCK” **OR** (ribonucleotide reductase M1 polypeptide) **OR** “ribosomal reductase M2” **OR** (ribonucleotide reductase M2 subunit*) **OR “**RRM2 protein” **OR (**ribonucleotide reductase M2 polypeptide) **OR** “ribonucleotide reductase M2 B (TP53 inducible) protein” **OR (**p53-inducible ribonucleotide reductase small subunit 2*) **OR “**NT5C3A” **OR (**Cytidine Deaminas*) **OR** “CDA” **OR** “ATP-Binding Cassette, Sub-Family B, Member 1” **OR** (P Glycoprotein*) **OR** (PGY 1 Protein*) **OR** “Multidrug Resistance Protein 1” **OR** “ABCB1 Protein” **OR** “MDR1 Protein” **OR** “rs1045642” **OR** “rs2032582” **OR** “rs1128503” **OR** “SLC22A12 protein” **OR (**urate transporter 1 protein *) **OR** “organic anion transpoter 4 like protein” **OR** (solute carrier family 22 organic anion cation transporters, member 12 protein*) **OR** “rs11231825” **OR (**NOS3 protein*) **OR** “nitric oxide synthase 3, endothelial cell protein, human” **OR** “ECNOS protein” **OR** “rs1799983” **OR** (Cytochrome P 450 CYP2E1*) **OR** (Cytochrome P 450 J*) **OR** (4 Nitrophenol 2 Hydroxylase*) **OR** (Dimethylnitrosamine N Demethylase*) **OR** “CYP 2E1” **OR** (Cytochrome P 450 IIE1*) **OR** (CYPIIE1*) **OR** “Cytochrome P-450 (ALC)” **OR** “CYP2E1” **OR** “rs2070673” **OR** “rs2515641”) |
| **#3** | TITLE-ABS-KEY (“randomized controlled trial” **OR**  “controlled clinical trial” [**OR** “randomized controlled trials” **OR** “random allocation” **OR** “double blind method” **OR**“single blind method”**OR** “clinical trial” **OR** “clinical trials” **OR** "cohort studies"**OR** “Concurrent Studies” **OR** “Closed Cohort Studies” **OR** “Cohort Analysis” **OR** “Historical Cohort Studies” **OR** “case-control studies”**OR** “Case-Control Study” **OR** “Case Comparison Studies” **OR** “Case-Compeer Study” **OR** “Case-Referrent Study” **OR** “Case Referrent Studies” **OR** “Case-Referrent Study” **OR** “Case-Base Studies” **OR** “Case Base Studies”**OR**“Case Control Studies” **OR** “Case Control Study” **OR** “Nested Case Control Studies” **OR** “Nested Case-Control Study”**OR** “Matched Case-Control Studies” **OR** “Matched Case-Control Study”) |
| **#4** | TITLE-ABS-KEY ((disease-free survival*)**OR** (Event-Free Survival*) **OR** (Progression-Free Survival*) **OR** " overall survival " **OR** “Drug Related Side Effects and Adverse Reactions” [MeSH Terms] **OR** “Adverse Drug Event” **OR** “Adverse Drug Events” **OR** “Drug Event, Adverse” **OR** “Drug Events, Adverse” **OR** “Side Effects of Drugs” **OR** “Drug Side Effects” **OR** “Drug Side Effect” **OR** “Effects, Drug Side” **OR** “Side Effect, Drug” **OR** “Side Effects, Drug” **OR** “Adverse Drug Reaction” **OR** “Adverse Drug Reactions” **OR** “Drug Reaction, Adverse” **OR** “Drug Reactions, Adverse” **OR** “Reactions, Adverse Drug” **OR** “Drug Toxicity” **OR** “Toxicity, Drug” **OR** “Drug Toxicities” **OR** “Toxicities, Drug” **OR** “overall response rate” **OR** “complete response” **OR** “complete response with incomplete blood recovery”) |
| **#5** | *SEARCH (COMBINE QUERIES)*  **#1** AND **#2** AND **#3** AND **#4** |
| **#6** | **Limits:** Species (humans), language (English); without limitation of age or year of publication. |
