# Supplementary material for: Evaluation of the impact of single-nucleotide polymorphisms on treatment response, survival and toxicity with cytarabine and anthracyclines in patients with acute myeloid leukaemia: a systematic review protocol
Source: Syst Rev. 2019 May 3;8:109. doi: 10.1186/s13643-019-1011-y (PMC6499963; doi:10.1186/s13643-019-1011-y)
Supplement: Supplementary file 5 — Web of Science database. (DOCX 16 kb) [file 13643_2019_1011_MOESM5_ESM.docx]

**Additional file 5.** Description of the search terms according to the Web of Science database.

|  | **Data base**: Web of Science  **Descriptors** |
| --- | --- |
| **#1** | TS(“Acute Myeloid Leukaemi*”) **OR** TS(“Acute Myeloid Leukemi*”) **OR** TS(“ANLL”) **OR** TS(“Leukaemia, Acute Myelogenous*”) **OR** TS(“Leukaemia, Acute Myeloid”) **OR** TS(“Leukaemia, Acute Myeloblastic”) **OR** TS(“Leukaemia, Acute Myelocytic”) **OR** TS(“Leukaemia, Acute Nonlymphoblastic”) **OR** TS(“Leukaemia, Acute Nonlymphocytic”) **OR** TS(“Leukaemias, Acute Myelogenous”) **OR** TS(“Leukaemias, Acute Myeloblastic”) **OR** TS(“Leukaemias, Acute Myelocytic”) **OR** TS(“Leukaemias, Acute Nonlymphoblastic”) **OR** TS(“Leukaemias, Acute Nonlymphocytic”) **OR** TS(“Myeloid Leukaemia, Acute, M1”) **OR** TS(“Acute Myeloid Leukaemia without Maturation”) **OR** TS(“Myeloid Leukaemia, Acute, M2”) **OR** TS(“Acute Myeloid Leukaemia with Maturation”) |
| **#2** | TS(“Single nucleotide polymorphism*”) **OR** TS(“SNPs”) **OR** TS(“rs2291075”) **OR** TS(“rs4149056”) **OR** TS(“[rs2306744](https://www.pharmgkb.org/variant/PA166156554)”) **OR** TS(**“**rs1042919”) **OR** TS(rs1561876”) **OR** TS(“rs1130609”) **OR** TS(**“**rs3750117) **OR** TS(**“**rs532545”) **OR** TS(“rs2072671”) **OR** TS(“Solute Carrier Organic Anion Transporter Family Member 1b1*”) **OR** TS(“SLC21A6 Transporter*”) **OR** TS(“LST-1 Transport Protein*”) **OR** TS(“Organic Anion Transport Polypeptide C*”) **OR** TS**(“**Oatp C Transport Protein*”) **OR** TS(**“**SLCO1B1 Protein”) **OR** TS**(“**Organic Anion Transport Polypeptide 2”) **OR** TS(“Deoxycytidine Kinase*”) **OR** TS(“DCK”) **OR** TS(“ribonucleotide reductase M1 polypeptide*”) **OR** TS(“ribosomal reductase M2”) **OR** TS(“ribonucleotide reductase M2 subunit*”) **OR** TS(**“**RRM2 protein”) **OR** TS**(“**ribonucleotide reductase M2 polypeptide”) **OR** TS(“ribonucleotide reductase M2 B (TP53 inducible) protein”) **OR** TS**(“**p53-inducible ribonucleotide reductase small subunit 2*”) **OR** TS(**“**NT5C3A”) **OR** TS**(“**Cytidine Deaminas*”) **OR** TS(“CDA”) **OR** TS(“ATP-Binding Cassette, Sub-Family B, Member 1”) **OR** TS(“P Glycoprotein*”) **OR** TS(“PGY 1 Protein*”) **OR** TS(“Multidrug Resistance Protein 1”) **OR** TS(“ABCB1 Protein”) **OR** TS(“MDR1 Protein”) **OR** TS(“rs1045642”) **OR** TS(“rs2032582”) **OR** TS(“rs1128503”) **OR** TS(“SLC22A12 protein”) **OR** TS**(“**urate transporter 1 protein*”) **OR** TS(“organic anion transpoter 4 like protein”) **OR** TS(”solute carrier family 22 organic anion cation transporters, member 12 protein*”) **OR** TS(“rs11231825”) **OR** TS**(“**NOS3 protein*”) **OR** TS(“nitric oxide synthase 3, endothelial cell protein, human”) **OR** TS(“ECNOS protein”) **OR** TS(“rs1799983”) **OR** TS(“Cytochrome P 450 CYP2E1*”) **OR** TS(“Cytochrome P 450 J*”) **OR** TS(“4 Nitrophenol 2 Hydroxylase*”) **OR** TS(“Dimethylnitrosamine N Demethylase*”) **OR** TS(“CYP 2E1”) **OR** TS(“Cytochrome P 450 IIE1*”) **OR** TS(“CYPIIE1*”) **OR** TS(“Cytochrome P-450 (ALC)”) **OR** TS(“CYP2E1”) **OR** TS(“rs2070673”) **OR** TS(“rs2515641”) |
| **#3** | TS(“randomized controlled trial”)**OR**TS( “controlled clinical trial”)**OR**TS (randomized controlled trials”)**OR**TS(“random allocation”)**OR**TS(“double blind method”)**OR**TS(“single blind method”)**OR**TS(“clinical trial”)**OR**TS(“clinical trials”)**OR** TS("cohort studies")**OR** TS(“Concurrent Studies”) **OR** TS(“Closed Cohort Studies”) **OR** TS(“Cohort Analysis”) **OR** TS(“Historical Cohort Studies”) **OR** TS(“case-control studies”)**OR** TS(“Case-Control Study”) **OR** TS(“Case Comparison Studies”) **OR** TS(“Case-Compeer Study”) **OR** TS(“Case-Referrent Study”) **OR** TS(“Case Referrent Studies”) **OR** TS(“Case-Referrent Study”) **OR** TS(“Case-Base Studies”) **OR** TS(“Case Base Studies”) **OR**TS(“Case Control Studies”) **OR** TS(“Case Control Study”) **OR** TS(“Nested Case Control Studies”) **OR** TS(“Nested Case-Control Study”) **OR** TS(“Matched Case-Control Studies”) **OR** TS(“Matched Case-Control Study”) |
| **#4** | TS(“disease-free survival*”) **OR** TS(“Event-Free Survival*”) **OR** TS(“Progression-Free Survival*”) **OR** TS("overall survival") **OR** TS(“Drug Related Side Effects and Adverse Reactions”) **OR** TS(“Adverse Drug Event”) **OR** TS(“Adverse Drug Events”) **OR** TS(“Drug Event, Adverse”) **OR** TS(“Drug Events, Adverse”) **OR** TS(“Side Effects of Drugs”) **OR** TS(“Drug Side Effects”) **OR** TS(“Drug Side Effect”) **OR** TS(“Effects, Drug Side”) **OR** TS(“Side Effect, Drug”) **OR** TS(“Side Effects, Drug”) **OR** TS(“Adverse Drug Reaction”) **OR** TS(“Adverse Drug Reactions”) **OR** TS(“Drug Reaction, Adverse”) **OR** TS(“Drug Reactions, Adverse”) **OR** TS(“Reactions, Adverse Drug”) **OR**  TS(“Drug Toxicity”) **OR** TS(“Toxicity, Drug”) **OR** TS(“Drug Toxicities”) **OR** TS(“Toxicities, Drug”) **OR** TS(“overall response rate”) **OR** TS(“complete response”) **OR** TS(“complete response with incomplete blood recovery”) |
| **#5** | **#1** AND **#2** AND **#3** AND **#4** |
| **#6** | **Limits:** Species (humans), language (English); without limitation of age or year of publication. |
