# Supplementary material for: Evaluation of the impact of single-nucleotide polymorphisms on treatment response, survival and toxicity with cytarabine and anthracyclines in patients with acute myeloid leukaemia: a systematic review protocol
Source: Syst Rev. 2019 May 3;8:109. doi: 10.1186/s13643-019-1011-y (PMC6499963; doi:10.1186/s13643-019-1011-y)
Supplement: Supplementary file 6 — Cochrane Clinical Trials database. (DOCX 17 kb) [file 13643_2019_1011_MOESM6_ESM.docx]

**Additional file 6**. Description of the search terms according to the Cochrane Clinical Trials database

|  | **Data base**: Cochrane Clinical Trials  **Descriptors** |
| --- | --- |
| **#1** | (“Acute Myeloid Leukaemi*”):ti,ab,kw **OR** (“Acute Myeloid Leukemi*”):ti,ab,kw **OR** (“ANLL”):ti,ab,kw **OR** (“Leukaemia, Acute Myelogenous*”):ti,ab,kw **OR** (“Leukaemia, Acute Myeloid”):ti,ab,kw **OR** (“Leukaemia, Acute Myeloblastic”):ti,ab,kw **OR** (“Leukaemia, Acute Myelocytic”):ti,ab,kw **OR** (“Leukaemia, Acute Nonlymphoblastic”):ti,ab,kw **OR** (“Leukaemia, Acute Nonlymphocytic”):ti,ab,kw **OR** (“Leukaemias, Acute Myelogenous”):ti,ab,kw **OR** (“Leukaemias, Acute Myeloblastic”):ti,ab,kw **OR** (“Leukaemias, Acute Myelocytic”):ti,ab,kw **OR** (“Leukaemias, Acute Nonlymphoblastic”):ti,ab,kw **OR** (“Leukaemias, Acute Nonlymphocytic”):ti,ab,kw **OR** (“Myeloid Leukaemia, Acute, M1”):ti,ab,kw **OR** (“Acute Myeloid Leukaemia without Maturation”):ti,ab,kw **OR** (“Myeloid Leukaemia, Acute, M2”):ti,ab,kw **OR** (“Acute Myeloid Leukaemia with Maturation”):ti,ab,kw |
| **#2** | (“Single nucleotide polymorphism*”):ti,ab,kw **OR** (“SNPs”):ti,ab,kw **OR** (rs2291075”):ti,ab,kw **OR** (“rs4149056”):ti,ab,kw **OR** (“[rs2306744](https://www.pharmgkb.org/variant/PA166156554)”):ti,ab,kw **OR** (**“**rs1042919”):ti,ab,kw **OR** (rs1561876”):ti,ab,kw **OR** (“rs1130609”):ti,ab,kw **OR** (**“**rs3750117):ti,ab,kw **OR** (**“**rs532545”):ti,ab,kw **OR** (“rs2072671”):ti,ab,kw **OR** (“Solute Carrier Organic Anion Transporter Family Member 1b1*”):ti,ab,kw **OR** (“SLC21A6 Transporter*”):ti,ab,kw **OR** (“LST-1 Transport Protein*”):ti,ab,kw **OR** (“Organic Anion Transport Polypeptide C*”):ti,ab,kw **OR (“**Oatp C Transport Protein*”):ti,ab,kw **OR** (**“**SLCO1B1 Protein”):ti,ab,kw **OR (“**Organic Anion Transport Polypeptide 2”):ti,ab,kw **OR** (“Deoxycytidine Kinase*”):ti,ab,kw **OR** (“DCK”):ti,ab,kw **OR** (“ribonucleotide reductase M1 polypeptide*”):ti,ab,kw **OR** (“ribosomal reductase M2”):ti,ab,kw **OR** (“ribonucleotide reductase M2 subunit*”):ti,ab,kw **OR** (**“**RRM2 protein”) :ti,ab,kw **OR (“**ribonucleotide reductase M2 polypeptide”):ti,ab,kw **OR** (“ribonucleotide reductase M2 B (TP53 inducible) protein”):ti,ab,kw **OR (“**p53-inducible ribonucleotide reductase small subunit 2*”):ti,ab,kw **OR** (**“**NT5C3A”):ti,ab,kw **OR (“**Cytidine Deaminas*”):ti,ab,kw **OR** (“CDA”):ti,ab,kw **OR** (“ATP-Binding Cassette, Sub-Family B, Member 1”):ti,ab,kw **OR** (“P Glycoprotein*”):ti,ab,kw **OR** (“PGY 1 Protein*”):ti,ab,kw **OR** (“Multidrug Resistance Protein 1”):ti,ab,kw **OR** (“ABCB1 Protein”):ti,ab,kw **OR** (“MDR1 Protein”):ti,ab,kw **OR** (“rs1045642”):ti,ab,kw **OR** (“rs2032582”):ti,ab,kw **OR** (“rs1128503”):ti,ab,kw **OR** (“SLC22A12 protein”):ti,ab,kw **OR (“**urate transporter 1 protein*”):ti,ab,kw **OR** (“organic anion transpoter 4 like protein”):ti,ab,kw **OR** (”solute carrier family 22 organic anion cation transporters, member 12 protein*”):ti,ab,kw **OR** (“rs11231825”):ti,ab,kw **OR (“**NOS3 protein*”):ti,ab,kw **OR** (“nitric oxide synthase 3, endothelial cell protein, human”):ti,ab,kw **OR** (“ECNOS protein”):ti,ab,kw **OR** (“rs1799983”):ti,ab,kw **OR** (“Cytochrome P 450 CYP2E1*”):ti,ab,kw **OR** (“Cytochrome P 450 J*”):ti,ab,kw **OR** (“4 Nitrophenol 2 Hydroxylase*”):ti,ab,kw **OR** (“Dimethylnitrosamine N Demethylase*”):ti,ab,kw **OR** (“CYP 2E1”):ti,ab,kw **OR** (“Cytochrome P 450 IIE1*”):ti,ab,kw **OR** (“CYPIIE1*”):ti,ab,kw **OR** (“Cytochrome P-450 (ALC)”):ti,ab,kw **OR** (“CYP2E1”):ti,ab,kw **OR** (“rs2070673”):ti,ab,kw **OR** (“rs2515641”):ti,ab,kw |
| **#3** | (“randomized controlled trial”):pt **OR**( “controlled clinical trial”):pt **OR** me(randomized controlled trials”) **OR** me(“random allocation”)**OR** me(“double blind method”) **OR** (“single blind method”) **OR** (“clinical trial”):pt **OR** me(“clinical trials”) **OR** ("cohort studies"):pt **OR** (“Concurrent Studies”):ti,ab,kw **OR** (“Closed Cohort Studies”):ti,ab,kw **OR** (“Cohort Analysis”):ti,ab,kw **OR** (“Historical Cohort Studies”):ti,ab,kw **OR** (“case-control studies”):ti,ab,kw **OR** (“Case-Control Study”) :ti,ab,kw **OR** (“Case Comparison Studies”) :ti,ab,kw **OR** (“Case-Compeer Study”) :ti,ab,kw **OR** (“Case-Referrent Study”):ti,ab,kw **OR** (“Case Referrent Studies”):ti,ab,kw **OR** (“Case-Referrent Study”):ti,ab,kw **OR** (“Case-Base Studies”):ti,ab,kw **OR** (“Case Base Studies”):ti,ab,kw **OR** (“Case Control Studies”):pt **OR** me(“Case Control Study”) **OR** (“Nested Case Control Studies”):ti,ab,kw **OR** (“Nested Case-Control Study”):ti,ab,kw **OR** (“Matched Case-Control Studies”):ti,ab,kw **OR** (“Matched Case-Control Study”):ti,ab,kw |
| **#4** | (“disease-free survival*”):ti,ab,kw **OR** (“Event-Free Survival*”):ti,ab,kw **OR** (“Progression-Free Survival*”):ti,ab,kw **OR** ("overall survival"):ti,ab,kw **OR** (“Drug Related Side Effects and Adverse Reactions”):ti,ab,kw **OR** (“Adverse Drug Event”):ti,ab,kw **OR** (“Adverse Drug Events”):ti,ab,kw **OR** (“Drug Event, Adverse”):ti,ab,kw **OR** (“Drug Events, Adverse”):ti,ab,kw **OR** (“Side Effects of Drugs”):ti,ab,kw **OR** (“Drug Side Effects”):ti,ab,kw **OR** (“Drug Side Effect”):ti,ab,kw **OR** (“Effects, Drug Side”):ti,ab,kw **OR** (“Side Effect, Drug”):ti,ab,kw **OR** (“Side Effects, Drug”):ti,ab,kw **OR** (Adverse Drug Reaction”):ti,ab,kw **OR** (“Adverse Drug Reactions”):ti,ab,kw **OR** (“Drug Reaction, Adverse”):ti,ab,kw **OR** (“Drug Reactions, Adverse”):ti,ab,kw **OR** (“Reactions, Adverse Drug”):ti,ab,kw **OR** (“Drug Toxicity”):ti,ab,kw **OR** (“Toxicity, Drug”):ti,ab,kw **OR** (“Drug Toxicities”):ti,ab,kw **OR**  (“Toxicities, Drug”):ti,ab,kw **OR** (“overall response rate”):ti,ab,kw **OR** (“complete response”):ti,ab,kw **OR** (“complete response with incomplete blood recovery”):ti,ab,kw |
| **#5** | **#1** AND **#2** AND **#3** AND **#4** |
| **#6** | **Limits:** Species (humans), language (English); without limitation of age or year of publication. |
