# Supplementary material for: Evaluation of the impact of single-nucleotide polymorphisms on treatment response, survival and toxicity with cytarabine and anthracyclines in patients with acute myeloid leukaemia: a systematic review protocol
Source: Syst Rev. 2019 May 3;8:109. doi: 10.1186/s13643-019-1011-y (PMC6499963; doi:10.1186/s13643-019-1011-y)
Supplement: Supplementary file 8 — Data dictionary for extract data. (DOCX 27 kb) [file 13643_2019_1011_MOESM8_ESM.docx]

**Additional file 8.** Date dictionary for extract data

|  | **Category PICOS** | **Variable** | **Content** | **Codes** |
| --- | --- | --- | --- | --- |
| 1 | Study Profile | reference | First author and date of publication | ex: Valter et al 2016 |
| 2 |  | study_type | Type of study evaluated | 1- Randomized Clinical Trial |
|  |  |  |  | 2- Cohort Study |
|  |  |  |  | 3- Control Case Study |
|  |  |  |  | 4- Descriptive study |
| 3 |  | study_country | Country of study | Open |
| 4 |  | context | Context of the study | Ex: Hospital, clinic, primary data collection etc. |
| 5 | Population | n_study | number of participants | in numbers |
| 6 |  | quant_grup | number of groups | in numbers |
| 7 |  | range_age_sample | The age range of the study participants | Categories of age range, median and median age will be accepted |
| 8 |  | criteria_eligibility | Eligibility criteria set out in Article | Open question |
| 9 |  | sample_selection_method | How this study was sampled | Open question |
| 10 |  | treatment | medications in use | 1. Cytarabine |
|  |  |  |  | 2. Cytarabine + Anthracyclic |
|  |  |  |  | 3. Cytarabine + Anthracyclic + others medications |
| 11 |  | type_lma | type of acute myeloid leukemia evaluated (FAB) | Open question |
| 12 | Exposures* | snpx | name of the SNP | Open question, eg: rs2291075 |
| 13 |  | localizationx | in which region the SNP is located | Open question |
| 14 |  | genex | related gene | Open question |
| 15 |  | basesx | Altered nitrogen bases | Open question |
| 16 | Outcomes | toxicity | Assessment of toxicity | 0. No |
|  |  |  |  | 1. Yes |
| 17 |  | degree_toxicity | Degree of toxicity | 1 a 5 |
| 18 |  | type_toxicity | Assessment of toxicity type according to CTCAE | Open question. Ex: neutropenia. |
| 19 |  | os | mean or median of the months | Open question |
| 20 |  | dfs | mean or median of the months | Open question |
| 21 |  | resp_tto | Response rate evaluation: complete response; partial response; drug resistance | Open question |
| 22 | Resultados | description_narrative | Narrative description of study results | Open question |
| 23 |  | description_evaluation_quantity | As the prognostic factor-mean, median, total count | Open question |
| 24 |  | ci_association_snp | Confidence interval of the association between exposure and outcome | Open question |
| 25 |  | p_value_association_snp | Statistical significance value for association between exposure and rejection of descriptive studies | Open question |
| 26 | Results | outcome_ecr | Type of primary endpoint evaluated on ECR | 1-Overal survival |
|  |  |  |  | 2- Disease free survival |
|  |  |  |  | 3- Response to treatment |
| 27 |  | description_evaluation_os1 | Mean / Median survival of group 1 for overall survival | Open question |
| 28 |  | descriotion_evaluation_os2 | Average / Median survival of group 2 for overall survival | Open question |
| 29 |  | risk_estimated_os | Relative risk or Hazard Ration for overall survival | Open question |
| 30 |  | ci_evaluation_os | Confidence interval of association between exposure and outcome in overall survival | Open question |
| 31 |  | p_value_os | Value of statistical significance for association between exposure and outcome in the overall survival assessment | Open question |
| 32 |  | description_evaluation_dfs1 | Mean / Median survival of group 1 for disease-free survival | Open question |
| 33 |  | description_evaluation_dfs2 | Mean / Median survival of group 2 for disease-free survival | Open question |
| 34 |  | risk_estimated_dfs | Relative risk and Hazard Ratio for evaluation of disease-free survival | Open question |
| 35 |  | ci_evaluation_dfs | Confidence interval of association between exposure and outcome in disease-free survival | Open question |
| 36 | Results | p_value_dfs | Value of statistical significance for association between exposure and free evaluation of disease - free survival. | Open question |
| 37 |  | description_evaluation_resp1 | Mean / Median answer rate of group 1 for drugs | Open question |
| 38 |  | description_evaluation_resp2 | Mean / Median answer rate of group 2 for drugs | Open question |
| 39 |  | risk_estimated_resp | Relative risk and Hazard Ratio for evaluation of response to treatment | Open question |
| 40 |  | ci_evaluation_resp | Confidence interval of association between exposure and outcome in response to treatment | Open question |
| 41 |  | p_value_resp | Value of statistical significance for association between exposure and free evaluation of disease - response to treatment | Open question |
| 42 |  | outcome_ cohort | Which outcome evaluated in the study | Open question |
| 43 | Results | description_evaluation_ cohort1 | Mean / Median / absolute frequency / relative frequency of outcome group 1 in the cohort | Open question |
| 44 |  | description_evaluation_ cohort2 | Mean / Median / absolute frequency / relative frequency of outcome group 2 in the cohort | Open question |
| 45 |  | risk_estimated_ cohort | Relative risk or Hazard Ration for outcome evaluation in the cohort | Open question |
| 46 |  | ci_ cohort | Confidence interval between exposure and outcome in the cohort | Open question |
| 47 |  | p_value_ cohort | Value of statistical significance for association between exposure and outcome in the cohort. | Open question |
| 48 |  | outcome_casoc | Which outcome was evaluated in the control case study. | Open question |
| 49 |  | description_evaluation_casoc1 | Mean / Median / absolute frequency / relative frequency of outcome group 1 in the control case. | Open question |
| 50 |  | description_evaluation_casoc2 | Mean / Median / absolute frequency / relative frequency of outcome group 2 in the control case. | Open question |
| 51 |  | risk_estimated_casoc | Relative risk or Hazard Ration for evaluation of the outcome in the control case. | Open question |
| 52 |  | ci_casoc | Confidence interval of the association between exposure and outcome in the control case | Open question |
| 53 |  | p_value_casoc | Value of statistical significance for association between exposure and unfavorable case control | Open question |

* An individual assessment will be done for each SNP evaluated in the manuscript.
